# The effects of Cumulative Trauma and Cognitive Rigidity on the Severity of Depressive Disorder: Preliminary Results

**DOI:** 10.1192/j.eurpsy.2022.1416

**Published:** 2022-09-01

**Authors:** M. Salla, M. Aguilera, C. Paz, J. Moya-Higueras, G. Feixas

**Affiliations:** 1 Universitat de Barcelona, Department Of Clinical Psychology And Psychobiology, BARCELONA, Spain; 2 Universitat de Barcelona, Department Of Cognition, Development And Educational Psychology, BARCELONA, Spain; 3 Universidad de Las Américas, School Of Psychology, Quito, Ecuador; 4 Universitat de Lleida, Department Of Psychology, Lleida, Spain

**Keywords:** traumatic events, dichotomous thinking, Depression

## Abstract

**Introduction:**

The long-lasting effects of trauma on mental health and the cumulative effect during the lifetime is one of the great interest in research and applied psychology. However, the effect of cumulative trauma in combination with cognitive biases, such as cognitive rigidity (“all-or-nothing” thinking pattern), on the severity of depression has not been tested yet.

**Objectives:**

The aim of this study was to analyse these variables, while considering for differential gender effects on a sample of patients with the diagnosis of depressive disorder.

**Methods:**

A total sample of 177 patients (137 women) were assessed using the Cumulative Trauma Scale. Cognitive rigidity was measured with the Repertory Grid Technique and severity of depressive symptoms with the Beck Depression Inventory.

**Results:**

indicated that high levels of cognitive rigidity and high frequency of perceived negative cumulative trauma predicted depressive symptoms; while high frequency of perceived positive trauma did not predict depressive symptoms. Moreover, gender did not explain variability of depression, and its interaction with frequency of perceived trauma was not significant.

**Conclusions:**

Overall, traumatic cumulative trauma frequency and its negative appraisal are key to the understanding the severity of depression but also cognitive rigidity seemed to be a relevant factor to consider. Thus, these results highlight the need to focus on traumatic and cognitive aspects to increase the efficacy of psychological interventions in depression.

**Disclosure:**

No significant relationships.

